# Triglyceride-glucose index and common carotid artery intima-media thickness in patients with ischemic stroke

**DOI:** 10.1186/s12933-022-01472-1

**Published:** 2022-03-18

**Authors:** Mengyuan Miao, Guo Zhou, Anran Bao, Yaming Sun, Huaping Du, Liyan Song, Yongjun Cao, Shoujiang You, Chongke Zhong

**Affiliations:** 1 Department of Epidemiology, School of Public Health and Jiangsu Key Laboratory of Preventive and Translational Medicine for Geriatric Diseases, Medical College of Soochow University, 199 Renai Road, Industrial Park District, Suzhou, 215123 Jiangsu China; 2 Department of Neurology and Suzhou Clinical Research Center of Neurological Disease, The Second Affiliated Hospital of Soochow University, Suzhou, China; 3 Department of Cardiology, Nantong Third People’s Hospital, Nantong University, Nantong, 226006 China; 4 Department of Neurology, Zhangjiagang TCM Hospital Affiliated to Nanjing University of Chinese Medicine, Suzhou, 215600 China; 5 Department of Neurology, The Affiliated Wujiang Hospital of Nantong University, Suzhou, 215200 China; 6 Department of Neurology, The First People’s Hospital of Taicang, Suzhou, 215400 China

**Keywords:** Triglyceride glucose index, Carotid artery intima-media thickness, Atherosclerosis, Ischemic stroke

## Abstract

**Background:**

Triglyceride glucose (TyG) index was recently reported to be associated with an increased risk of the development and recurrence of cardiovascular events, and atherosclerosis is a main speculative mechanism. However, data on the relationship between TyG index and atherosclerosis, especially in the setting of ischemic stroke, is rare. We aimed to explore the association between TyG index and carotid atherosclerosis in patients with ischemic stroke.

**Methods:**

A total of 1523 ischemic stroke patients with TyG index and carotid artery imaging data were enrolled in this analysis. The TyG index was calculated as ln [fasting triglyceride (mg/dL) × fasting glucose (mg/dL)/2]. Carotid atherosclerosis was measured by common carotid artery intima-media thickness (cIMT), and abnormal cIMT was defined as a mean cIMT and maximum cIMT value ≥ 1 mm. Multivariable logistic regression models and restricted cubic spline models were used to assess the relationships between TyG index and abnormal cIMT. Risk reclassification and calibration of models with TyG index were analyzed.

**Results:**

The multivariable-adjusted odds ratios (95% CIs) in quartile 4 versus quartile 1 of TyG index were 1.56 (1.06–2.28) for abnormal mean cIMT and 1.46 (1.02–2.08) for abnormal maximum cIMT, respectively. There were linear relationships between TyG index and abnormal mean cIMT (*P* for linearity = 0.005) and abnormal maximum cIMT (*P* for linearity = 0.027). In addition, the TyG index provided incremental predictive capacity beyond established risk factors, shown by an increase in net reclassification improvement and integrated discrimination improvement (all *P* < 0.05).

**Conclusions:**

A higher TyG index was associated with carotid atherosclerosis measured by cIMT in patients with ischemic stroke, suggesting that TyG could be a promising atherosclerotic marker.

## Introduction

Stroke is the leading cause of adult mortality and serious, long-term disability in China and is characterized by high rates of recurrent stroke and cardiovascular events [1]. Atherosclerosis is a main pathological basis of stroke development and recurrence [2, 3]. Thus, it is crucial to identify high‐risk patients early and timely control atherosclerosis progression.

Insulin resistance (IR) is a well-known predictor of a wide range of cardiovascular diseases (CVDs) [4–6]. Although the hyperinsulinemic-euglycemic clamp is the gold standard test for the measurement of IR, it is inappropriate in clinical settings due to the complex detection process and economic reasons. Therefore, markers of IR that are simple, stable and available to implement are urgently needed. Lately, the triglyceride glucose (TyG) index, the product of triglyceride (TG) and fasting plasma glucose (FPG), has been proposed as a reliable alternative marker of IR, with a close relationship with the gold-standard method [7]. Additionally, the TyG index has even been shown to predict IR in a better performance than surrogate markers such as homeostasis model assessment of IR (HOMA-IR) [8]. Growing evidence suggests that the TyG index is a promising predictive marker for CVD [9–12]. Recently, an elevated TyG index was reported to be associated with an increased risk of both stroke incidence and recurrence in Chinese populations [13–15]. The main speculative mechanism is atherosclerosis, however, population-based evidence on the relationship between TyG index and atherosclerosis has not yet been clearly established, especially in the setting of ischemic stroke.

Several previous studies have shown that an elevated TyG index was associated with an increased risk of subclinical atherosclerosis, including coronary artery calcium and carotid plaque [16, 17]. Common carotid artery intima-media thickness (cIMT), a noninvasive imaging marker of carotid atherosclerosis, can instantly identify early atherosclerotic vascular changes and has become a valuable tool for evaluating and monitoring the progression of CVD. In light of these findings, we hypothesized that a high TyG index was associated with an increased risk of atherosclerosis measured by cIMT.

To provide population-based evidence on the role of the TyG index in the progression of carotid atherosclerosis and enhance the understanding of the TyG index-stroke prognosis relationship, we therefore aimed to investigate whether the TyG index is associated with carotid atherosclerosis measured by cIMT in patients with ischemic stroke.

## Materials and methods

### Study participants

From December 2013 to May 2014, we consecutively recruited patients with acute ischemic stroke from 22 hospitals in Suzhou city, China. The diagnosis of ischemic stroke was made according to World Health Organization-defined criteria based on patient history, clinical characteristics, and neuroimaging CT or magnetic resonance imaging data of the brain. The exclusion criteria were as follows: (1) patients with a diagnosis of transient ischemic attack; (2) time from onset to admission over 7 days; (3) lack of carotid artery imaging data; and (4) lack of TG and FPG concentrations. Finally, 1523 ischemic stroke patients with available carotid artery imaging and TyG index data were included in this analysis.

### Data collection

Data on demographic characteristics, clinical features, medical history (hypertension, diabetes mellitus, stroke, atrial fibrillation and coronary heart disease), medication history (antihypertensive and lipid-lowering), and imaging data were collected at the time of enrollment. All information was obtained using a standard questionnaire by trained neurologists or nurses in participating hospitals. Stroke severity was evaluated by qualified neurologists using the National Institutes of Health Stroke Scale (NIHSS) at admission [18]. Cigarette smoking was defined as smoking at least one cigarette per day for more than one year. Alcohol consumption was defined as consuming at least 1 alcoholic drink per day in the past year. Three blood pressure measurements were obtained at baseline by trained nurses using a standard mercury sphygmomanometer according to the protocol adapted from procedures recommended by the American Heart Association [19]. Hypertension was defined as systolic blood pressure ≥ 140 mmHg and/or diastolic blood pressure ≥ 90 mmHg or the use of antihypertensive medications. Diabetes mellitus was defined as fasting glucose ≥ 7.0 mmol/L, nonfasting glucose ≥ 11.1 mmol/L with classic symptoms of hyperglycemia or hyperglycemic crisis, or use of glucose-lowering drugs. A history of atrial fibrillation was confirmed on ≥ 1 electrocardiogram or the presence of arrhythmia during hospitalization.

We collected blood samples at morning after at least 8 h of fasting within 24 h of hospital admission. Plasma glucose, triglyceride levels and other biochemical parameters were analyzed by automatic biochemical analyzer using fasting blood at local laboratories [20]. Glucose and triglyceride values were converted from mmol/L to mg/dL and multiplied by 18.020 and 88.545, respectively. The triglyceride-glucose index was calculated as ln [triglyceride (mg/dL) × fasting glucose (mg/dL)/2] [21].

### Carotid artery measurements

Carotid ultrasonography examinations were performed by certified sonographers who received unified training and were unaware of the baseline characteristics and laboratory results of the participants [22]. Following the Mannheim consensus, the region of interest for cIMT measurement was the far wall of bilateral common carotid arteries proximal to the bifurcation, along a plaque-free segment of ≥ 10 mm long at each side [23]. All participants were examined using high‐resolution B‐mode ultrasound systems. In each participating hospital, two standardized trained sonographers measured carotid artery imaging data, discrepancies between their evaluations were resolved by consensus, and the final consistent carotid artery imaging data between the two certified sonographers were recorded. Carotid artery imaging data showed good agreement between the two certified sonographers.

Both mean cIMT and maximum cIMT were used for analysis. Mean cIMT was defined as the mean value of the right and left common carotid arteries. Maximum cIMT was defined as the larger value of the right and left common carotid artery (i.e., if the right cIMT was larger than the left cIMT, the right cIMT value was used). Abnormal mean cIMT and maximum cIMT were defined as the mean cIMT value and maximum cIMT value ≥ 1 mm [24, 25].

### Statistical analysis

All participants were stratified into quartiles based on their baseline calculated TyG index levels. Continuous variables are presented as the mean ± SD or median (25th-75th percentile), and categorical variables are expressed as frequencies (%). Generalized linear regression analysis was used to test for trends across TyG index quartiles for continuous variables, while the Cochran-Armitage trend χ^2^ test was used for categorical variables. Multiple imputation for missing covariates was performed using the Markov chain Monte Carlo method.

Logistic regression models were conducted to estimate the relationship between TyG index and carotid atherosclerosis. We calculated the odds ratios (ORs) and 95% confidence intervals (CIs) for the highest quartile of TyG index compared with the lowest quartile and for a 1 SD increment in TyG index levels. Tests for linear trends in ORs across TyG quartiles were conducted using the median within each quartile as the predictor. We performed 3 logistic regression models. Model 1 was an age- and gender-adjusted model; model 2 further adjusted for current smoking, alcohol consumption and hospitals; model 3 further adjusted for admission NIHSS score, systolic blood pressure, diastolic blood pressure, high-density lipoprotein, medical history (hypertension, diabetes mellitus, atrial fibrillation), use of antihypertensive medications and lipid-lowering medications, time from onset to hospital, and stroke syndrome. Furthermore, we conducted subgroup analyses to assess whether the potential covariables (age, gender, current smoking, alcohol consumption, admission NIHSS score, history of hypertension and diabetes) modified the relationship between TyG index and carotid atherosclerosis. The interactions between TyG index and covariates of interest were tested using the likelihood ratio test of models with interaction terms. Additionally, we applied restricted cubic spline regression models with 4 knots (at the 5th, 35th, 65th and 95th percentiles) to explore the shapes of the associations between TyG index and abnormal mean and maximum cIMT.

Furthermore, we tested the predictive value of TyG index for carotid atherosclerosis. The Hosmer–Lemeshow χ^2^ statistic was used to evaluate the calibration of models with TyG index. C statistics, the net reclassification index (NRI) and integrated discrimination improvement (IDI) were calculated to assess risk discrimination and reclassification by adding TyG index to the basic model with established risk factors.

All statistical tests were two-sided, and *P* values < 0.05 were considered statistically significant. Statistical analysis was conducted using SAS statistical software (version 9.4; SAS Institute, Cary, NC).

## Results

### Baseline characteristics

Our study included 1523 ischemic stroke patients with integrated TyG index and carotid artery imaging data. The mean age (SD) of the participants was 68.8 (12.2) years, and the median TyG index concentration was 8.69 (interquartile range, 8.28–9.19) mg/dL. The baseline characteristics of the study participants according to quartiles of TyG index are shown in Table 1. Compared with the lowest quartile of TyG index, participants with higher levels of TyG were more inclined to be younger; to have higher systolic blood pressure, diastolic blood pressure, TG, total cholesterol, low-density lipoprotein cholesterol, plasma glucose, and white blood cell count; to have a higher prevalence of hypertension and diabetes; to have a higher proportion of antihypertensive medication; to have a higher proportion of posterior circulation stroke syndrome and large-artery atherosclerosis stroke subtypes; and to be more inclined to have a lower level of high-density lipoprotein cholesterol and to have a lower proportion of atrial fibrillation and cardioembolism stroke subtypes.

**Table 1:** Baseline characteristics according to quartiles of TyG index

	TyG index					*P*
Characteristics	Total	< 8.28	8.28–8.69	8.69–9.19	≥ 9.19	trend
Demographic						
Age, year	68.8 ± 12.2	71.6 ± 12.9	69.8 ± 11.8	68.2 ± 11.8	65.5 ± 11.7	< 0.0001
Male sex	915 (60.1)	240 (63.2)	229 (60.0)	225 (58.6)	221 (58.6)	0.53
Current cigarette smoking	324 (21.3)	83 (21.8)	73 (19.1)	88 (22.9)	80 (21.2)	0.62
Current alcohol drinking	152 (10.0)	36 (9.5)	32 (8.4)	42 (10.9)	42 (11.1)	0.54
Clinical features						
Time from onset to randomization, h	24.0 (6.0–69.7)	24.0 (5.0–48.0)	24.0 (6.0–72.0)	24.0 (7.0–48.0)	24.0 (7.0–72.0)	0.005
Baseline SBP, mmHg	152.2 ± 22.1	149.7 ± 23.7	152.6 ± 21.0	153.5 ± 22.0	152.8 ± 21.5	0.047
Baseline DBP, mmHg	85.3 ± 12.5	83.5 ± 13.0	85.5 ± 12.2	85.9 ± 12.6	86.2 ± 11.9	0.003
Triglyceride, mmol/L	1.3 (0.9–1.8)	0.7 (0.6–0.9)	1.1 (1.0–1.3)	1.5 (1.3–1.8)	2.4 (1.7–3.2)	< 0.0001
Total cholesterol, mmol/L	4.6 (3.9–5.3)	4.3 (3.5–4.9)	4.5 (3.9–5.2)	4.7 (4.1–5.4)	5.1 (4.2–5.9)	< 0.0001
LDL cholesterol, mmol/L	2.7 (2.2–3.4)	2.4 (1.9–2.9)	2.7 (2.2–3.3)	2.9 (2.3–3.5)	2.9 (2.4–3.6)	< 0.0001
HDL cholesterol, mmol/L	1.2 (1.0–1.4)	1.3 (1.1–1.5)	1.2 (1.0–1.4)	1.1 (1.0–1.3)	1.0 (0.9–1.2)	< 0.0001
Plasma glucose, mmol/L	6.8 ± 3.7	5.0 ± 0.9	5.7 ± 1.2	6.4 ± 1.6	10.0 ± 6.1	< 0.0001
White blood cell count, 10^9^/L	6.6 (5.4–8.2)	6.3 (5.0–7.8)	6.6 (5.4–8.2)	6.8 (5.7–8.3)	6.9 (5.8–8.5)	0.01
Admission NIHSS score	3.0 (2.0–6.0)	4.0 (2.0–7.0)	3.0 (2.0–5.0)	3.0 (2.0–6.0)	3.0 (2.0–5.0)	0.17
Medical history						
Hypertension	1215 (79.8)	272 (71.6)	310 (81.2)	311 (81.0)	322 (85.4)	< 0.0001
Diabetes	397 (26.1)	26 (6.8)	53 (13.9)	98 (25.5)	220 (58.4)	< 0.0001
Coronary heart disease	70 (4.6)	16 (4.2)	12 (3.1)	21 (5.5)	21 (5.6)	0.33
Atrial fibrillation	196 (12.9)	83 (21.8)	54 (14.1)	30 (7.8)	29 (7.7 )	< 0.0001
Stroke	1201 (78.9)	293 (77.1)	304 (79.6)	306 (79.7)	298 (79.1)	0.8
Medication history						
Antihypertensive	895 (58.8)	191 (50.3)	228 (59.7)	239 (62.2)	237 (62.9)	0.001
Lipid-lowering	46 (3.0)	17 (4.5)	9 (2.4)	14 (3.7)	6 (1.6)	0.09
Stroke syndrome						
TACS	82 (5.4)	29 (7.6)	17 (4.5)	14 (3.7)	22 (5.8)	0.08
PACS	798 (52.4)	215 (56.6)	197 (51.6)	204 (53.1)	182 (48.3)	0.14
POCS	379 (24.9)	65 (17.1)	89 (23.3)	108 (28.1)	117 (31.0)	< 0.0001
LACS	264 (17.3)	71 (18.7)	79 (20.7)	58 (15.1)	56 (14.9)	0.09
Ischemic stroke subtype						
Large-artery atherosclerosis	949 (62.3)	193 (50.8)	225 (58.9)	253 (65.9)	278 (73.7)	< 0.0001
Cardioembolism	254 (16.7)	107 (28.2)	66 (17.3)	44 (11.5)	37 (9.8)	< 0.0001
Lacunar	215 (14.1)	50 (13.2)	67 (17.5)	54 (14.1)	44 (11.7)	0.12
Other	105 (6.9)	30 (7.9)	24 (6.3)	33 (8.6)	18 (4.8)	0.16

Continuous variables are expressed as mean ± SD or as median (interquartile range);

Categorical variables are expressed as frequencies (percentages)

*TyG index* Triglyceride glucose index, *SBP* systolic blood pressure, *DBP* diastolic blood pressure, *cIMT* carotid artery intima-media thickness, *HDL* high-density lipoprotein, *LDL* low-density lipoprotein, *NIHSS* National Institutes of Health Stroke Scale, *TACS* total anterior circulation Syndrome, *PACS* partial anterior circulation syndrome, *POCS* posterior circulation syndrome, and *LACS* lacunar syndrome

### Triglyceride-glucose index and cIMT

The relationships between TyG index and carotid atherosclerosis are presented in Table 2. In the age- and gender-adjusted logistic regression models, compared with the lowest quartile, the ORs (95% CIs) for the highest quartile of TyG index were 1.54 (1.10–2.15) for abnormal mean cIMT and 1.52 (1.11–2.08) for abnormal maximum cIMT, respectively. After further adjustment for covariables in model 2, a higher TyG index remained significantly associated with an increased risk of abnormal cIMT. Moreover, the relationships between TyG index and abnormal cIMT were still significant even after adjustment for potential confounding factors (model 3). The fully adjusted ORs (95% CIs) in quartile 4 versus quartile 1 were 1.56 (1.06–2.28) for abnormal mean cIMT (*P* for trend = 0.03) and 1.46 (1.02–2.08) for abnormal maximum cIMT (*P* for trend = 0.046) (Table 2).

**Table 2:** Odds ratios (95% CIs) of abnormal cIMT according to quartiles of TyG index

	TyG index				*P*	Each 1-SD
	< 8.28	8.28–8.69	8.69–9.19	≥ 9.19	trend	increase in TyG index
Median	8.01	8.49	8.89	9.62		
Abnormal mean cIMT						
Cases, n (%)	107 (28.2)	114 (29.8)	104 (27.1)	114 (30.2)		
Model 1	1.00	1.23 (0.89–1.70)	1.16 (0.84–1.62)	1.54 (1.10–2.15)	0.02	1.17 (1.06–1.30)
Model 2	1.00	1.24 (0.90–1.71)	1.17 (0.84–1.63)	1.56 (1.12–2.19)	0.01	1.18 (1.06–1.30)
Model 3	1.00	1.24 (0.89–1.73)	1.16 (0.83–1.64)	1.56 (1.06–2.28)	0.03	1.18 (1.05–1.33)
Abnormal maximum cIMT						
Cases, n (%)	137 (36.1)	141 (36.9)	136 (35.4)	146 (38.7)		
Model 1	1.00	1.16 (0.85–1.57)	1.17 (0.86–1.60)	1.52 (1.11–2.08)	0.009	1.15 (1.05–1.27)
Model 2	1.00	1.16 (0.86–1.58)	1.18 (0.86–1.60)	1.53 (1.12–2.10)	0.008	1.15 (1.04–1.23)
Model 3	1.00	1.15 (0.84–1.57)	1.15 (0.83–1.58)	1.46 (1.02–2.08)	0.046	1.14 (1.02–1.27)

Abnormal mean and maximum cIMT were defined as cIMT value ≥ 1 mm;

To convert triglyceride values from mmol/L to mg/dL, multiply by 88.545;

To convert plasma glucose values from mmol/L to mg/dL, multiply by 18.020;

Model 1: adjusted for age, gender;

Model 2: further adjusted for current smoking, alcohol consumption, and hospital;

Model 3: further adjusted for admission NIHSS score, SBP, DBP, high-density lipoprotein, medical history (hypertension, diabetes mellitus, atrial fibrillation), use of antihypertensive medications and lipid-lowering medications, time from onset to hospital, and stroke syndrome.

*CI* confidence interval, *cIMT* carotid artery intima-media thickness, *TyG* triglyceride-glucose index

**Fig. 1:**
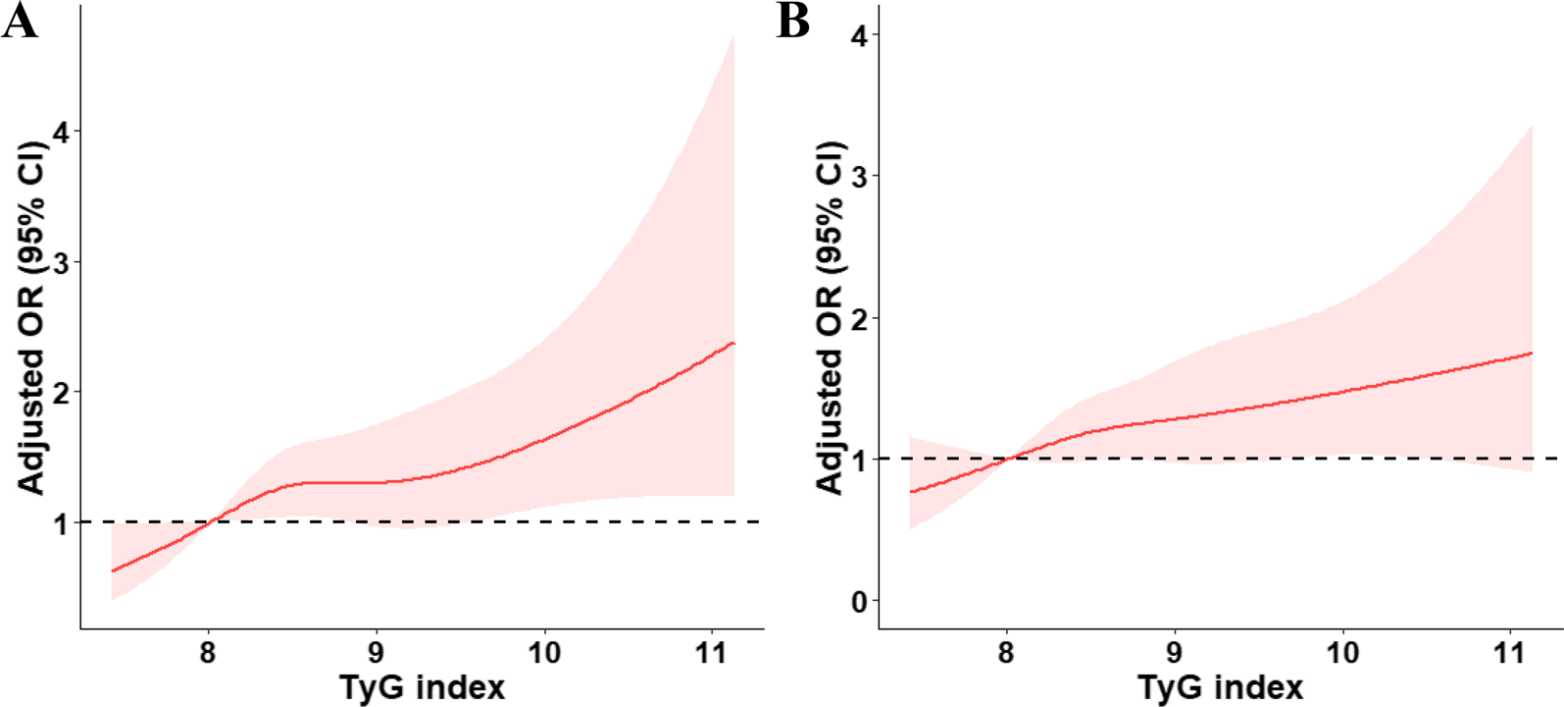
Relationship of Triglyceride-glucose Index and Common Carotid Artery Intima-Media Thickness in Patients with Ischemic Stroke. Odds ratios and 95% confidence intervals derived from restricted cubic spline regression, with knots placed at the 5th, 35th, 65th, and 95th percentiles of the distribution of triglyceride-glucose index levels. The reference point is the midpoint (8.01 mg/dL) of the reference group from categorical analysis. Odds ratios were adjusted for the same variables as model 3 in Table 2. cIMT indicates carotid artery intima-media thickness. Panel A: Abnormal mean cIMT (*P* for linearity = 0.005); Panel B: Abnormal maximum cIMT (*P* for linearity = 0.027). Abnormal mean and maximum cIMT were defined as cIMT values ≥ 1 mm

In addition, per 1-SD higher TyG index was associated with an 18% (95% CI: 5–33%) and a 14% (95% CI: 2–27%) increased risk of abnormal mean cIMT and abnormal maximum cIMT in fully adjusted model 3, respectively. Multiple-adjusted spline regression models showed linear relationships between TyG index and abnormal mean cIMT (*P* for linearity = 0.005; Fig. 1A) and abnormal maximum cIMT (*P* for linearity = 0.027; Fig. 1B).

The subgroup analyses indicated that TyG index was positively associated with carotid atherosclerosis in most categories, stratified by age, gender, current smoking, alcohol consumption, baseline NIHSS score, history of hypertension and history of diabetes. No significant interactions between TyG index and these potential atherosclerosis risk factors for interest were observed (all *P* for interaction > 0.05) (Table 3).

**Table 3:**
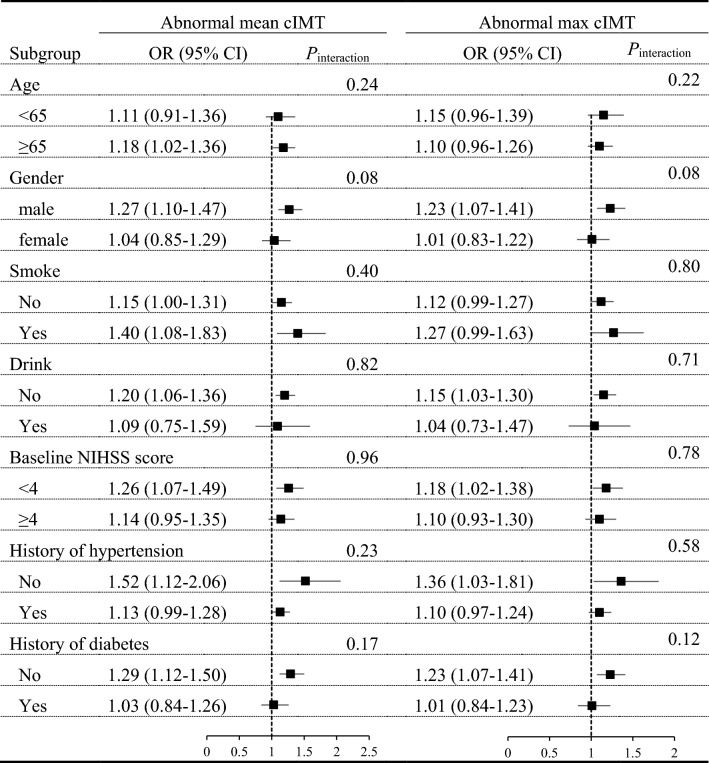
Subgroup analyses of the association between TyG index and abnormal cIMT

*OR* odds ratios, *CI* confidence interval, *NIHSS* National Institute of Health Stroke Scale, *TyG index* Triglyceride glucose index; ORs were adjusted for age, gender, current smoking, alcohol consumption, hospital, admission NIHSS score, SBP, DBP, high-density lipoprotein, medical history (hypertension, diabetes mellitus, atrial fibrillation), use of antihypertensive medications and lipid-lowering medications, time from onset to hospital, and stroke syndrome

### Incremental predictive value of TyG index

We further assessed the predictive ability of TyG index beyond established risk factors for carotid atherosclerosis measured by cIMT (Table 4). The basic model included age, gender, current smoking, alcohol consumption, hospital, admission NIHSS score, systolic blood pressure, diastolic blood pressure, high-density lipoprotein, medical history (hypertension, diabetes mellitus, atrial fibrillation), use of antihypertensive medications and lipid-lowering medications, time from onset to hospital, and stroke syndrome. First, the Hosmer Lemeshow test showed that the model calibrations were adequate after the addition of TyG index to the basic model (both *P* > 0.05). Second, the addition of TyG index to the basic model significantly improved risk classification for both abnormal mean cIMT (continuous-free NRI = 13.85%, 95% CI: 2.78–24.91%, *P* = 0.01; IDI = 0.53%, 95% CI: 0.15–0.91%, *P* = 0.007) and abnormal maximum cIMT (continuous-free NRI = 11.44%, 95% CI: 1.05%-21.84%, *P* = 0.03; IDI = 0.32%, 95% CI: 0.03–0.62%, *P* = 0.03). However, adding TyG index to the basic model did not significantly improve discriminatory power.

**Table 4:** Performance of models with TyG index to predict carotid atherosclerosis in patients with acute ischemic stroke

	Calibration		C statistic		NRI (continuous)		IDI	
	χ^2^	*P*	Estimate	*P*	Estimate	*P*	Estimate	*P*
		value	(95% CI), %	Value	(95% CI), %	value	(95% CI), %	value
Abnormal mean cIMT								
Basic model	13.3	0.1	0.647 (0.622–0.671)		Reference		Reference	
Basic model + TyG index	8.4	0.39	0.652 (0.628–0.676)	0.26	13.85 (2.78–24.91)	0.01	0.53 (0.15–0.91)	0.007
Abnormal maximum cIMT								
Basic model	1.9	0.98	0.634 (0.610–0.659)		Reference		Reference	
Basic model + TyG index	5.8	0.67	0.639 (0.615–0.663)	0.3	11.44 (1.05–21.84)	0.03	0.32 (0.03–0.62)	0.03

The basic model included age, gender, current smoking, alcohol consumption, hospital, admission NIHSS score, SBP, DBP, high-density lipoprotein, medical history (hypertension, diabetes mellitus, atrial fibrillation), use of antihypertensive medications and lipid-lowering medications, time from onset to hospital, and stroke syndrome

*CI* confidence interval, *TyG index* Triglyceride glucose index, *IDI* integrated discrimination improvement, *NRI* net reclassification index

## Discussion

In this multicenter population-based study of ischemic stroke, we found that a higher TyG index was associated with carotid atherosclerosis in patients with acute ischemic stroke, including abnormal mean cIMT and maximum cIMT. There was a positive dose-dependent relationship between TyG index and carotid atherosclerosis. Similar findings were observed in the subgroup analysis, further emphasizing the robustness of these associations. Moreover, the addition of TyG index to the traditional risk model significantly promoted the ability for risk stratification of abnormal mean cIMT and maximum cIMT. Our study demonstrated that TyG index may have an important implication for atherosclerosis prevention among patients with ischemic stroke.

Existing epidemiologic studies have shown that the TyG index was associated with major cardiovascular risk factors and an increased risk of various CVDs [12, 14, 26]. A large national representative cohort study of Koreans with 5,593,134 participants suggested that TyG index is an independent predictor of myocardial infarction and stroke [12]. The Kailuan cohort reported that elevated levels of the baseline and long-term TyG index were associated with an increased risk of myocardial infarction and ischemic stroke [14, 26]. In addition, a meta-analysis of eight cohort studies suggested that a higher TyG index may be independently associated with a higher incidence of atherosclerosis CVD, coronary artery disease, and stroke [27]. Furthermore, previous cross-sectional and retrospective evidence has shown that TyG index was also associated with an increased risk of subclinical atherosclerosis as assessed by coronary artery calcium, plaque, arterial stiffness and stenoses, which is suggested to be the underlying mechanism of stroke development and recurrence [28–31].

The cIMT assessed by ultrasonography of carotid arteries is a feasible, noninvasive, and accurate method for detecting early signs of atherosclerosis, which may reflect the diverse biological characteristics of atherosclerotic phenomena [32]. Moreover, cIMT has been extensively used for evaluating cardiovascular risk in primary prevention settings and several pathophysiologic conditions [33–35]. The I-Lan Longitudinal Aging Study (ILAS) demonstrated that a higher TyG index was significantly associated with abnormally elevated cIMT in nondiabetic women after adjusting for the traditional risk factors of atherosclerosis [36]. Our study enriches the limited current evidence on the association between TyG index and carotid atherosclerosis measured by cIMT and extends this relationship specifically to patients with ischemic stroke. We found a positive dose–response relationship between TyG index and abnormal cIMT in patients with ischemic stroke. Our findings together with previous evidence supported a close relationship between the TyG index and carotid atherosclerosis.

The mechanisms underlying the association of TyG index with carotid atherosclerosis have not been clearly elucidated, but it may be linked to IR. First, the TyG index is an alternative marker of IR, and prior studies suggested that IR could accelerate atherosclerosis progression by causing metabolic abnormalities, such as dyslipidemia, hypertension, and hyperglycemia [4, 37, 38]. IR was shown to interfere with insulin receptors and insulin receptor-mediated signaling at the level of atherosclerotic lesion cells, including macrophages, endothelial cells, and vascular smooth muscle cells [39, 40]. In addition, IR has been reported to be associated with chronic inflammation, which could be induced by various proinflammatory cytokines and oxidative stress biomarkers [41]. Second, the TyG index has also been considered as a suitable and reliable marker of systemic inflammation [42–44]. Furthermore, higher plasma TG levels were thought to promote endothelial dysfunction, plaque rupture and arterial inflammation [45], while elevated FPG levels could induce oxidative stress, alter protein kinase signaling, and trigger certain miRNA and epigenetic modifications [46], which were regarded as another possible pathogenesis in the atherosclerosis process. More studies are needed to clarify the mechanisms of TyG with a carotid atherosclerosis relationship.

To the best of our knowledge, this is the first report to investigate TyG index and the link to carotid atherosclerosis measured in patients with ischemic stroke. In the present study, rigid quality control procedures were used in data collection. Additionally, important confounders were also collected and controlled in the multivariable adjusted models, so our study could provide a more valid assessment of the effect of TyG index on carotid atherosclerosis. Our findings provide epidemiological evidence to illuminate the hypothesis that TyG causes atherosclerosis as a plausible mechanism for TyG index-stroke prognosis relationship, suggesting an important role for a high level of TyG index in the pathogenesis of atherosclerosis. The TyG index can be easily calculated by TG and FPG without additional cost and has a better performance for the prediction of IR. Therefore, the TyG index is expected to find wide application in clinical practice to identify patients at a higher risk of stroke incidence and recurrence early. Our study has important clinical implications for better understanding the pathogenesis and optimizing treatment strategies of ischemic stroke.

Our study also had several limitations as follows. First, our cross-sectional study cannot exclude the possibility of residual confounders, which limited us to establish a causal relation between TyG index and carotid atherosclerosis in patients with ischemic stroke. Therefore, future large-scale prospective studies are needed to verify the present findings. Second, we did not conduct serial measurements of TyG index; thus, we were unable to study the association between TyG index changes and cIMT in the setting of ischemic stroke. Third, we did not record the original data of the two sonographers, which limited us to calculate the coefficient of variation. However, in our study, all the sonographers received unified training and were unaware of the baseline characteristics and laboratory results of the participants. Moreover, all procedures were conducted according to Mannheim consensus. Finally, our participants were exclusively Chinese ischemic stroke patients and we also excluded patients with transient ischemic attack, so the generalizability of our findings might be a concern.

In conclusion, elevated levels of TyG index were associated with carotid atherosclerosis measured by cIMT among patients with ischemic stroke, even after adjusting for potential traditional risk factors. Our study indicated that this simple index may be useful for identifying individuals at high risk of recurrent cardiovascular events in the early stages of ischemic stroke.

## Data Availability

The datasets used and/or analyzed during the current study are available from the corresponding author on reasonable request.
